# Supplementary material for: Bile canaliculi remodeling activates YAP via the actin cytoskeleton during liver regeneration
Source: Mol Syst Biol. 2020 Feb 24;16(2):e8985. doi: 10.15252/msb.20198985 (PMC7036714; doi:10.15252/msb.20198985)
Supplement: Supplementary file 1 — Appendix [file MSB-16-e8985-s001.pdf]

Appendix

Bile canaliculi remodeling activates YAP via the actin cytoskeleton during liver regeneration

Kirstin Meyer[1], Hernan Morales-Navarrete[1], Sarah Seifert[1], Michaela Wilsch-Braeuninger[1], Uta Dahmen[2], Elly M. Tanaka[3], Lutz Brusch[5], Yannis Kalaidzidis[1,4] and Marino Zerial[1,*]

Max Planck Institute of Molecular Cell Biology and Genetics, Dresden, Saxony 01307, Germany

Experimental Transplantation Surgery, Department of General, Visceral and Vascular Surgery, Jena University Hospital, 07747 Jena, Germany

Research Institute of Molecular Pathology, Vienna Biocenter, 1030, Vienna, Austria

Faculty of Bioengineering and Bioinformatics, Moscow State University, 119991 Moscow, Russia

Center for Information Services and High Performance Computing, Technische Universität Dresden, 01062 Dresden, Germany

* Corresponding Author

Table of contents.

Appendix Figures - Pages 2 – 10

Appendix Tables – Pages 11 - 14

Supplemental Information – Pages 15 - 28

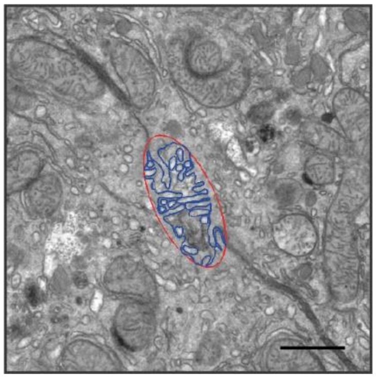

Figure S1 Determination of BC membrane length and perimeter from EM images
Representative EM image segmentation of the BC membrane (blue) and the minimal enclosing ellipse (perimeter) of that segmentation (red). Scale bar, 1 µm.

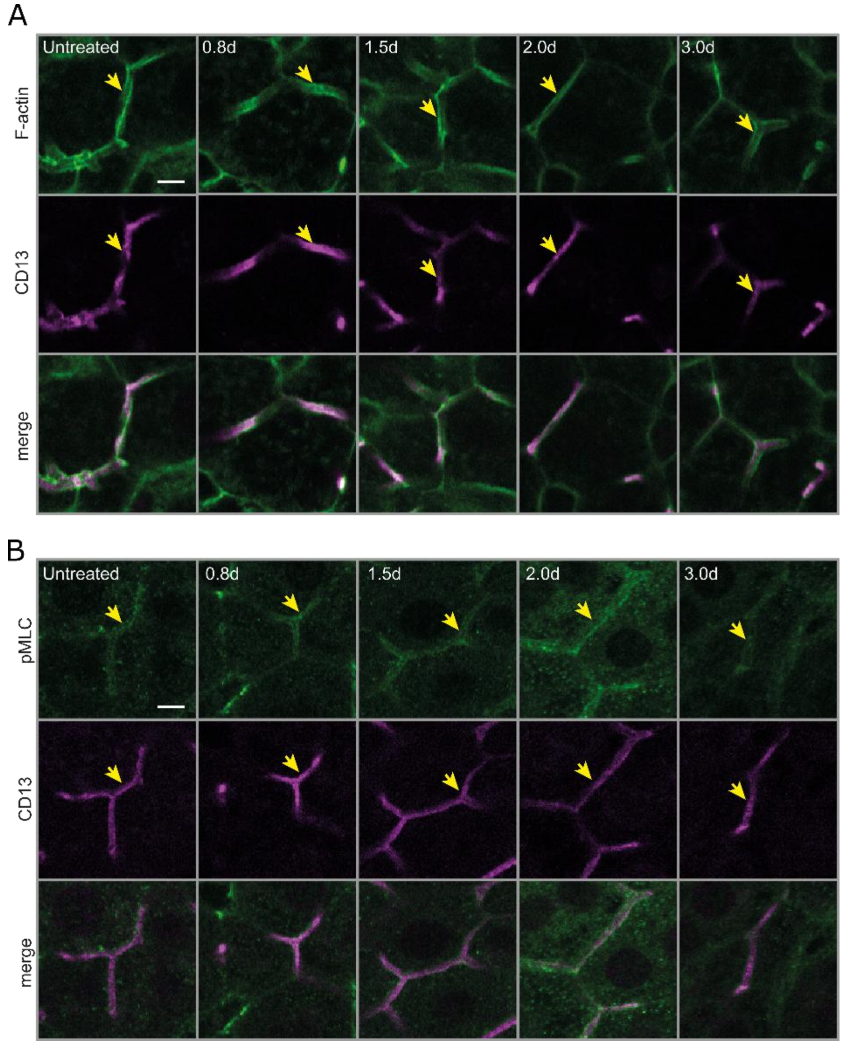

Figure S2 Fluorescence staining of apical F-actin and pMLC in livers after sham OP

A, B) Fluorescence stainings for F-actin (A) or pMLC (B) and the apical marker CD13 on liver tissue sections from untreated mice or animals at indicated time points post sham OP. Images were taken in the PV area. Arrows indicate BC. Scale bar, 5 µm (A, B).

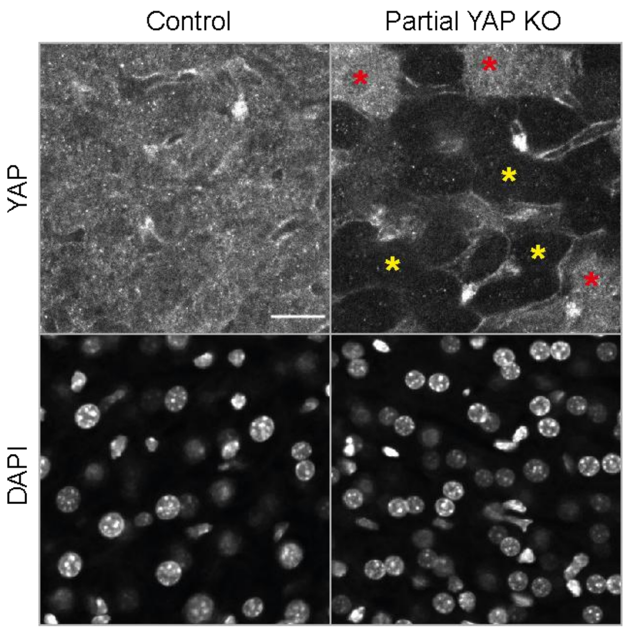

Figure S3 Validation of YAP antibody on liver tissue sections

Fluorescence staining for YAP and with DAPI on liver tissue sections from control or conditional partial YAP knockout (KO) mice. The knockout was specifically induced in hepatocytes by adeno-associated virus mediated expression of Cre-recombinase from an Albumin promotor (pALB) in YAP$^{fl/fl}$ mice. Yellow asterisks indicate KO hepatocytes, red asterisks indicate uninfected cells. Control mice received EGFP expressing adeno-associated-virus (EGFP expression not shown). Scale bar, 20 µm.

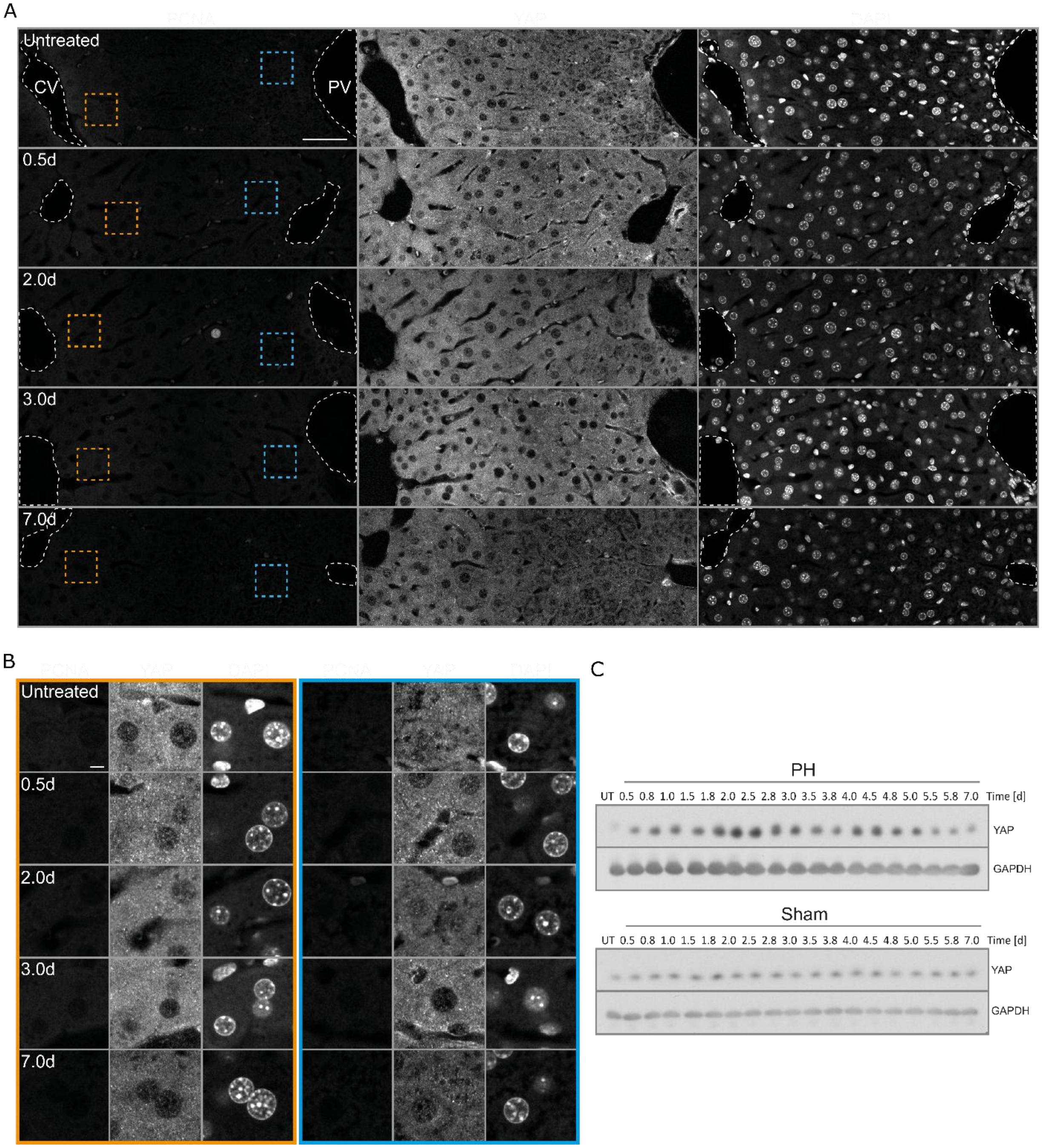

Figure S4 Immunofluorescence staining for YAP and PCNA on liver tissue sections after sham OP

A, B) Fluorescence stainings for YAP, PCNA and with the nuclear marker DAPI on liver tissue sections from untreated mice or animals at indicated time points post sham OP. Images show an entire CV-PV axis (CV, left; PV, right), veins are indicated by white dashed lines. Indicated regions (dashed rectangle) in the CV (orange) and PV (blue) area in (A) are shown as magnifications in (B). **C)** Western blot detection of YAP and GAPDH (loading control) in liver tissue lysates of untreated mice or animals at indicated time points post PH (upper panel) or sham OP (lower panel). UT, untreated control. Images in (A) and (B) are background-subtracted. Scale bars, 50 µm (A), 5 µm (B).

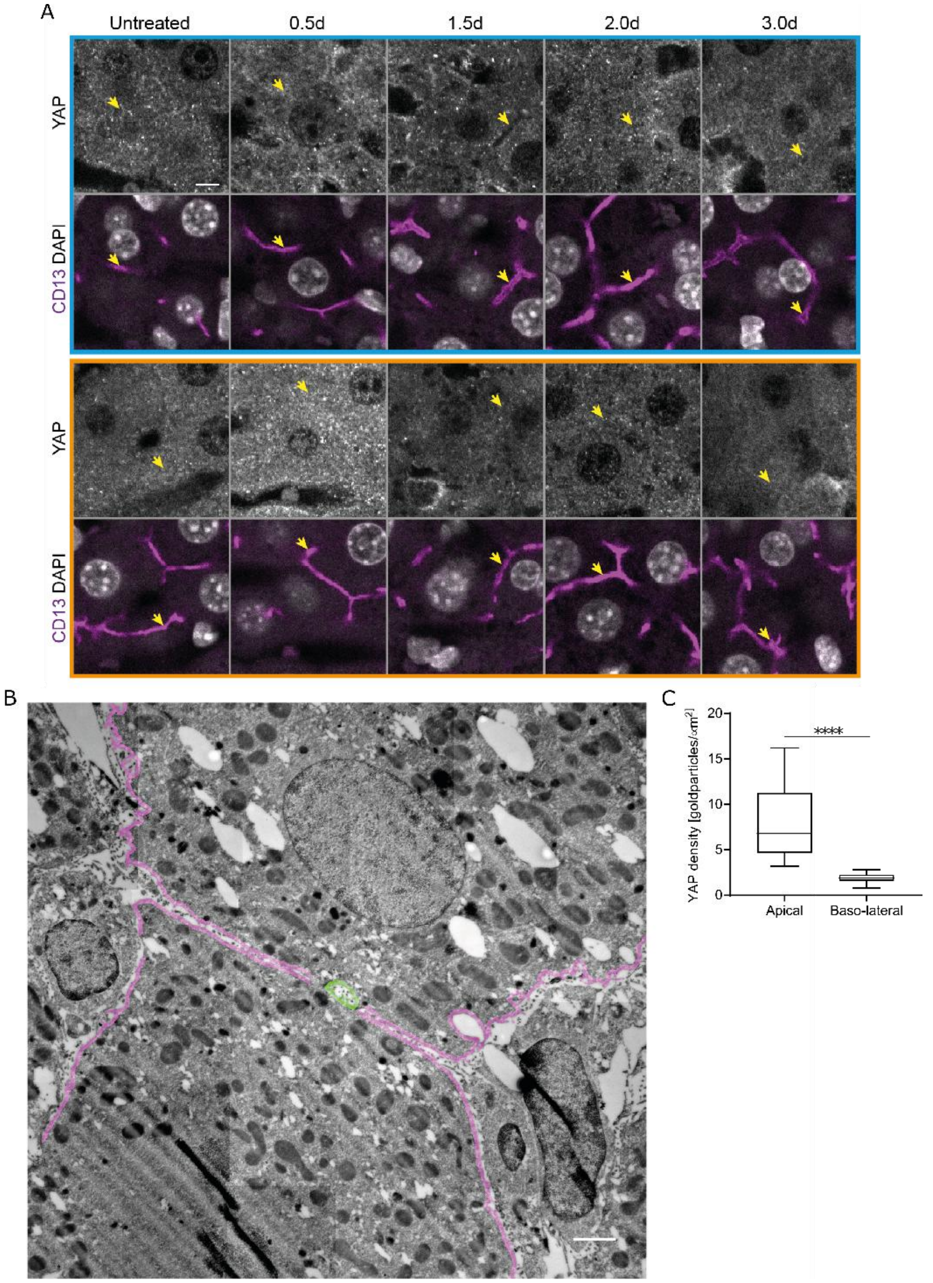

Figure S5 Analysis of apical YAP localization

A) Fluorescence stainings for YAP, CD13 and with the nuclear marker DAPI on liver tissue sections from untreated mice or animals at indicated time points post sham OP in the PV (blue, upper panel) and CV (orange, lower panel) area. Arrows indicate BC. **B)** Representative EM image showing segmentations of the sub-apical (green) and basolateral (magenta) area within a distance of 200 nm below the plasma membrane. **C)** Quantification of YAP density in the sub-apical or -basolateral membrane area (200 nm below the membrane) from YAP immuno-EM image grids on liver tissue sections at 1.5-1.8 d post PH as shown in (B). Box-whisker plot with median, 25-75 quartiles and minimum/maximum error bars, n=13 EM image grids from a total of 2 mice. Gold particle density in apical vs. basolateral area, $p=3.78*10^{-7}$. Images in (A) are background-subtracted. Scale bars, 5 µm (A), 2 µm (B).

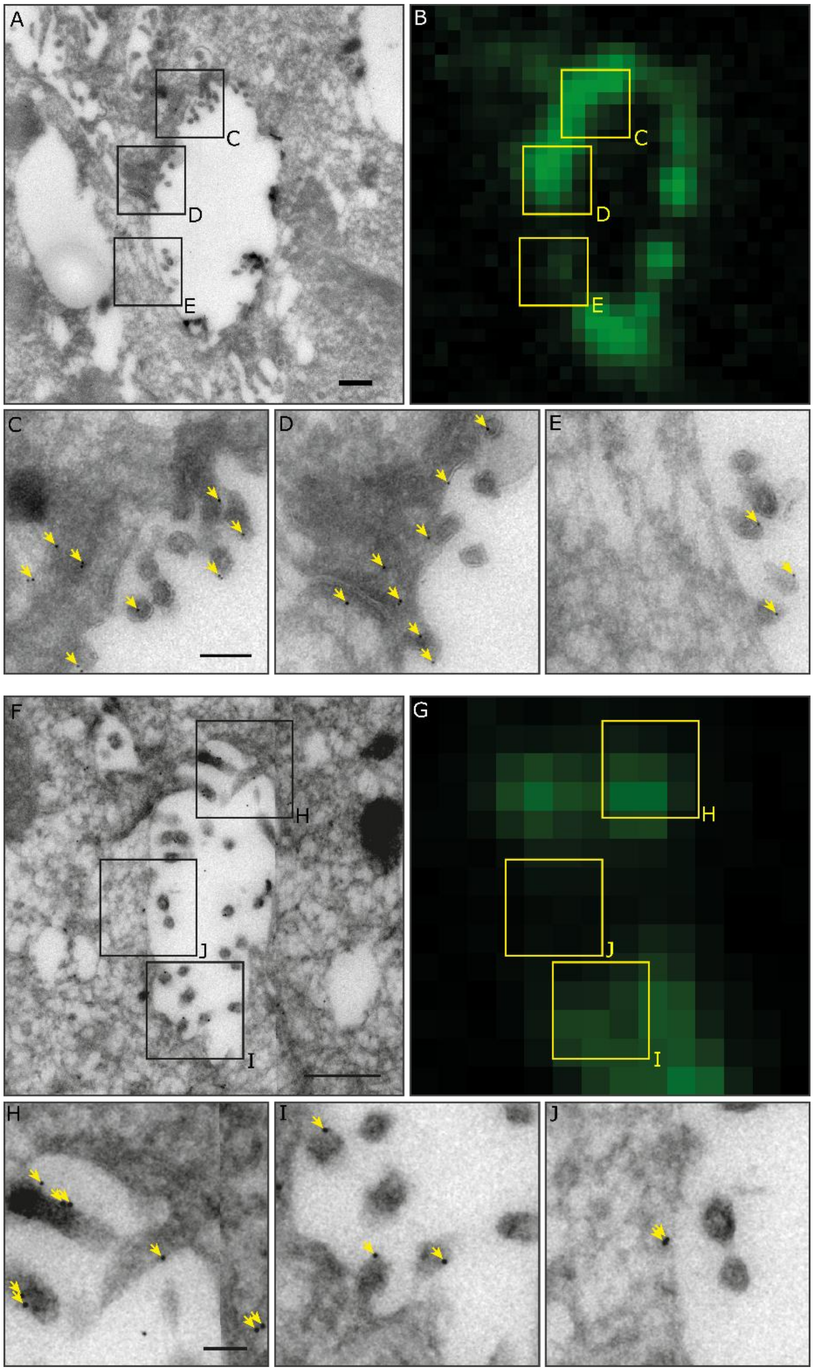

Figure S6 Correlative light and electron microscopy of F-actin and YAP on liver tissue sections during regeneration

A-E) Correlative light and electron microcopy of F-actin and YAP on a liver tissue section at 1.8 d post PH. YAP was detected by immunogold-labelling, F-actin by fluorescence staining with phalloidin. Shown are 2 BC (A, F), their respective F-actin staining (B, G) as well as magnifications of the indicated areas (black rectangle) with high (C, D and H, I) or low (E and J) F-actin levels. Arrows indicate gold particles. Images in (B) and (G) are background-subtracted. Scale bar, 0.5 µm (A, F), 0.2 µm (C), 0.1 µm (H).

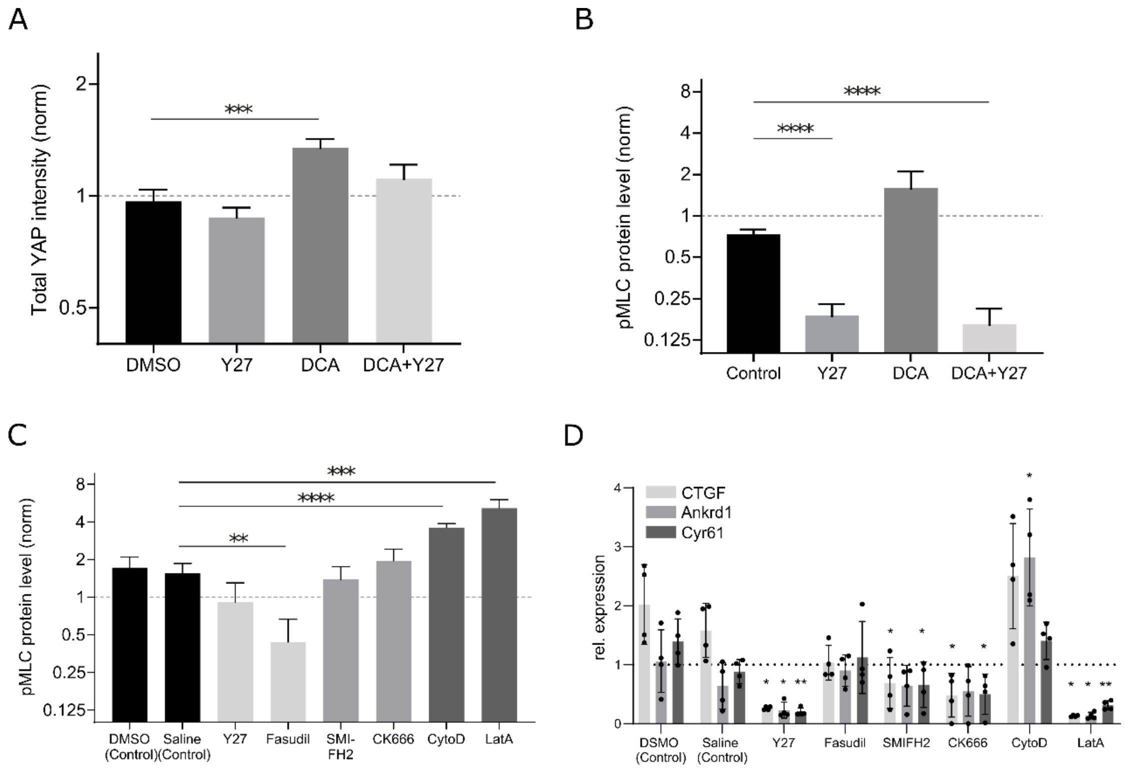

Figure S7 Quantification of YAP and pMLC levels in primary hepatocyte cultures upon treatment with DCA and actin inhibitors

A) Quantification of the mean cellular YAP levels in control (DMSO) and Y27, DCA and DCA+Y27 treated hepatocyte cultures. For full description of conditions, see legend of Fig.5B. Data was normalized to untreated condition (not shown). Shown are mean ± s.e.m, n= 7. Total YAP intensity of DMSO vs. DCA treated cells, p=0.001; DMSO vs. DCA+Y27, p=0.25 (n.s.); DMSO vs. Y27, p=0.34 (n.s.); DCA vs. DCA+Y27, p=0.07 (n.s.). **B**) Quantification of pMLC in primary hepatocyte culture lysates from Western blot as representatively shown in Fig.5C. Cells were incubated for ~18 h with the indicated compounds. Data is normalized to untreated cells (not shown). Mean ± s.e.m., n = 6. pMLC protein levels of DMSO vs. Y27, $p=3.15*10^{-11}$; DMSO vs. DCA+Y27, $p=5.88*10^{-11}$; DMSO vs. DCA, p = 0.11 (n.s.). **C**) Quantification of pMLC in actin-inhibitor treated primary hepatocyte culture lysates from Western blots as representatively shown in Fig.5F. Cells were treated with DMSO, Saline, Y27, Fasudil, SMIFH2, CK666, CytoD or Latrunculin A (LatA) for 6 h. Saline serves as control for Fasudil, DMSO serves as control for all other conditions. Data is normalized to untreated cells (not shown). Mean ± s.e.m., n = 5. pMLC protein level of DMSO vs. CytoD treated cells, $p=3.60*10^{-5}$; DMSO vs. LatA, p=0.0003; Saline vs. Fasudil, p=0.004; DMSO vs. Y27, p=0.13 (n.s.); DMSO vs. SMIFH2, p=0.51 (n.s.); DMSO vs. CK666, p= 0.70 (n.s.). **D**) qPCR analysis of YAP target gene (Cyr61, CTGF, Ankrd1) expression in hepatocyte culture upon actin inhibitor treatment as described in panel (C). Expression levels are normalized to an untreated control (not shown). Mean ± s.e.m., n = 4. Significant differences (compared to control condition) are indicated by asterisks: *, p ≤ 0.05; **, p < 0.01. DMSO vs. Y27, p=0.01 (CTGF), p=0.05 (Ankrd1), p=0.01 (Cyr61); DMSO vs. SMIFH2, p=0.02 (CTGF), p=0.04 (Cyr61); DMSO vs. CK666, p=0.01 (CTGF), p=0.01 (Cyr61); DMSO vs. CytoD, p=0.02 (Ankrd1); DMSO vs. LatA, p=0.01 (CTGF), p=0.04 (Ankrd1), p=0.01 (Cyr61).

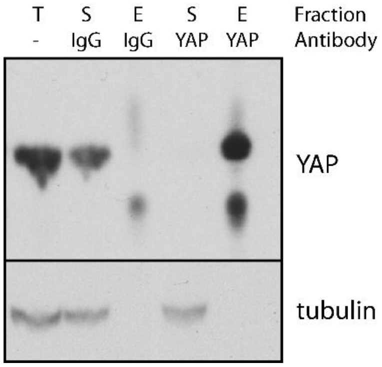

Figure S8 Co-IP of YAP from regenerating liver tissue

Co-IP of YAP from regenerating liver at 1.5d post PH. Shown is a Western blot of YAP and tubulin (loading control) in the total lysate (T), supernatant (S) and eluate (E) fraction of the co-IP. Immunoprecipitation with IgG was used as control for unspecific binding of proteins, while a YAP specific antibody was used for IP of YAP interactors.

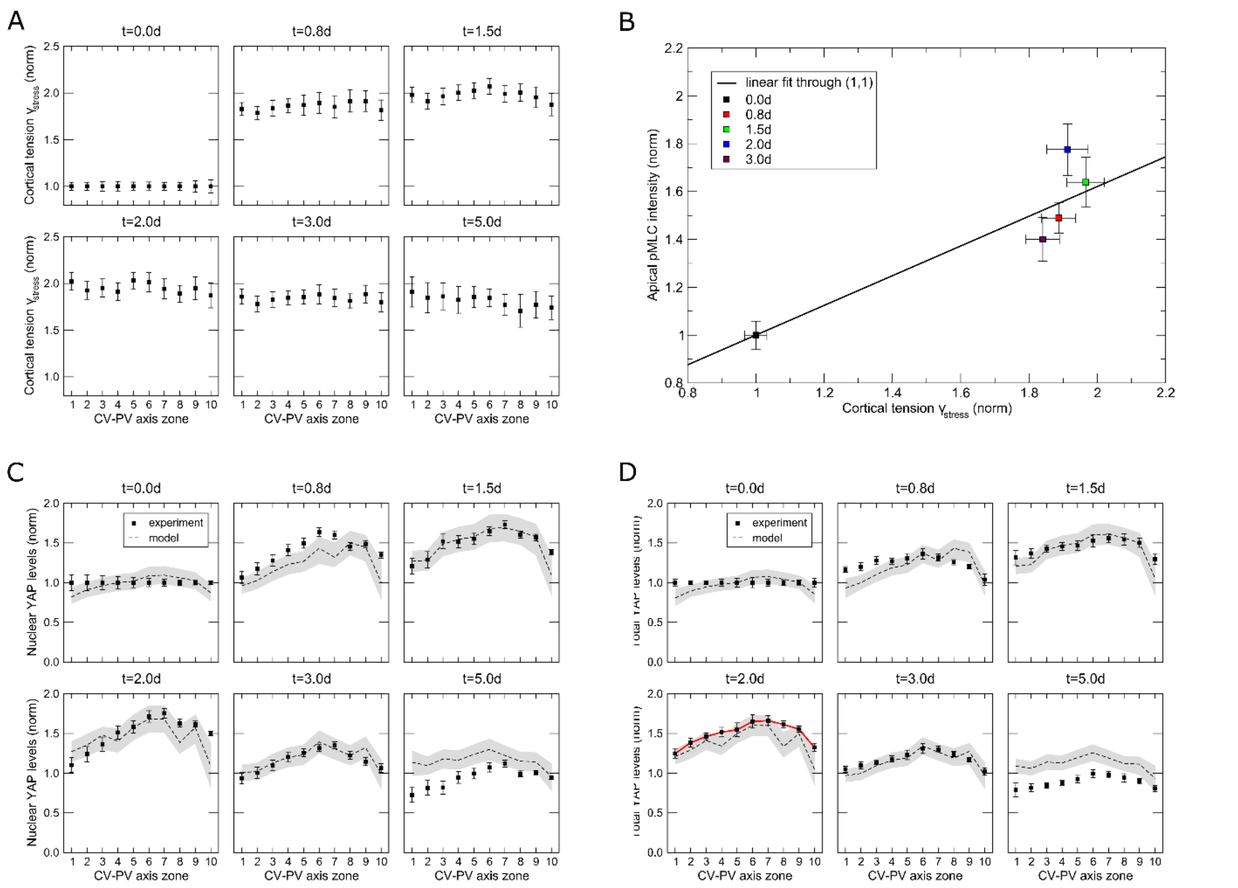

Figure S9 Prediction of cortical tension and YAP behavior during regeneration

A) Spatial profiles of predicted cortical tension during regeneration. Shown are predictions of the normalized mean ± s.e.m. cortical tension within 10 zones between the CV (zone 1) and PV (zone 10) axis at indicated timepoints (0.8 – 5.0 d post PH). The untreated condition is denoted as timepoint 0.0 d. The input data from Fig. 1B at 18 spatial positions was interpolated to align with the 10 positions of data from Fig. 3D. **B**) Correlation between predicted cortical tension and measured apical pMLC levels at different time points post PH (see legend) and in the untreated liver (t = 0.0 d). The apical pMLC intensity is derived from data reported in Fig. 2D and cortical tension from panel (A) is averaged over the corresponding positions in the PV area. The diagonal line represents the linear curve fit, see text. **C, D**) Comparison between the predicted (dashed black line) and measured (symbols) norm. nuclear (C) or total (D) YAP levels within 10 zones between the CV (zone 1) and PV (zone 10) axis at indicated timepoints (0.8 – 5.0 d) post PH and in the untreated liver (0.0 d). Shown are mean ± s.e.m. (error bar or grey corridor). In (D), the model input *s(x)* is shown as red curve (connecting the means of measured data). Experimental data in (C) is reproduced from Fig. 3D.

Appendix Tables

Table S1 Proteomic analysis of YAP interactors during regeneration

NCBI accession number	Protein (Gene symbol)	Abundance (norm)
568958510	yes-associated protein1(Yap1)	1
110431378	urotrophin(Utrn)	0.991889321
26344914	tyrosine 3-monooxygenase/tryptophan 5-monooxygenase activation protein, epsilon polypeptide(Ywhae)	0.387677036
148676868	tyrosine 3-monooxygenase/tryptophan 5-monooxygenase activation protein, zeta polypeptide(Ywhaz)	0.333398925
124487317	itchy, E3 ubiquitin protein ligase(Itch)	0.254979348
3065929	tyrosine 3-monooxygenase/tryptophan 5-monooxygenase activation protein, gamma polypeptide(Ywhag)	0.23426012
50510881	cingulin(Cgn)	0.210475673
124487163	kinesin family member 13B(Kif13b)	0.170951632
568940758	membrane associated guanylate kinase, WW and PDZ domain containing 1(Magi1)	0.165974153
7710096	syntrophin, basic 1(Sntb1)	0.162731866
6756037	tyrosine 3-monooxygenase/tryptophan 5-monooxygenase activation protein, eta polypeptide(Ywhah)	0.142245328
148702066	mCG7879	0.134723345
254675277	tight junction protein 1(Tjp1)	0.121933703
190359883	membrane associated guanylate kinase, WW and PDZ domain containing 3(Magi3)	0.107408584
590121980	angiomotin(Amot)	0.105382927
148671596	mCG22338	0.097859075
11558398	WW domain containing transcription regulator 1(Wwtr1)	0.067419449
124339826	heat shock protein 1B(Hspa1b)	0.066725689
13386106	nudix (nucleoside diphosphate linked moiety X)-type motif 21(Nudt21)	0.066147197
114050335	WW, C2 and coiled-coil domain containing 1(Wwc1)	0.062732151
256665243	cleavage and polyadenylation specific factor 7(Cpsf7)	0.05862306
568963046	angiomotin-like 2(Amotl2)	0.057292382
568924220	ubiquitin-associated protein 2-like(Ubap2l)	0.054917833
109730945	agmatine ureohydrolase (agmatinase)(Agmat)	0.051214475
1374782	neural precursor cell expressed, developmentally down-regulated 4(Nedd4)	0.049190786
148694057	uveal autoantigen with coiled-coil domains and ankyrin repeats(Uaca)	0.044790766
112799851	transformation related protein 53 binding protein 2(Trp53bp2)	0.03940942
148705099	mCG10028	0.037268682
6678059	syntrophin, basic 2(Sntb2)	0.03570625
1872343	anti-DNA immunoglobulin heavy chain IgG	0.033854384
22122593	HAUS augmin-like complex, subunit 1(Haus1)	0.033275733
269954704	HAUS augmin-like complex, subunit 4(Haus4)	0.027719949
148709667	tight junction protein 2(Tjp2)	0.027604438
1167906	alpha-1(XVIII) collagen	0.024479357
226442837	HAUS augmin-like complex, subunit 5(Haus5)	0.021759221

568935978	septin 11(Sept11)	0.020486408
148708896	peroxiredoxin 4(Prdx4)	0.020196788
568991034	TRIO and F-actin binding protein(Triobp)	0.019907355
148669446	dystrobrevin, beta(Dtnb)	0.01892376
134053862	MAGI family member, X-linked(Magix)	0.018634355
164519057	polycystic kidney disease 2(Pkd2)	0.018634355
148678168	diaphanous related formin 1(Diaph1)	0.01765076
133778926	enabled homolog (Drosophila)(Enah)	0.017361327
148666661	MOB kinase activator 1A(Mob1a)	0.017361327
154146185	capping protein regulator and myosin 1 linker 1(Carmil1)	0.017071707
755533730	septin 10(Sept10)	0.017071707
254939680	SMAD specific E3 ubiquitin protein ligase 2(Smurf2)	0.01678249
148699206	mCG15824	0.01678249
22122693	HAUS augmin-like complex, subunit 3(Haus3)	0.016203624
568945325	protein phosphatase 1, regulatory (inhibitor) subunit 12C(Ppp1r12c)	0.015798895
13994195	protein phosphatase 1, catalytic subunit, alpha isoform(Ppp1ca)	0.015219842
46559772	HAUS augmin-like complex, subunit 6(Haus6)	0.014930624
74184100	unnamed protein product	0.013946842
568928294	PATJ, crumbs cell polarity complex component(Patj)	0.013367976
568972723	syntaxin binding protein 4(Stxbp4)	0.013367976
124487107	angiomotin-like 1(Amotl1)	0.013078758
148689890	cleavage and polyadenylation specific factor 6(Cpsf6)	0.012094976
568926128	multiple PDZ domain protein(Mpdz)	0.012094976
755524534	Rho guanine nucleotide exchange factor (GEF7)(Arhgef7)	0.010243111
115270958	GTPase, very large interferon inducible 1(Gvin1)	0.010243111
114052811	tight junction protein 3(Tjp3)	0.009953678
9625023	membrane protein, palmitoylated 5 (MAGUK p55 subfamily member 5)(Mpp5)	0.008680678
116174774	Sec24 related gene family, member A (S. cerevisiae)(Sec24a)	0.008391245
21361647	adenosylhomocysteinase like 1(AHCYL1)	0.008391245
161086971	capping protein (actin filament) muscle Z-line, alpha 1(Capza1)	0.008101831
148690623	TEA domain family member 3(Tead3)	0.007118094
19526848	mitochondrial amidoxime reducing component 2(Marc2)	0.007118094
110625853	zinc finger, FYVE domain containing 1(Zfyve1)	0.006828812
19882245	family with sequence similarity 122, member A(Fam122a)	0.006828812
22094997	HAUS augmin-like complex, subunit 7(Haus7)	0.006828812
112181167	complement component 1, q subcomponent binding protein(C1qbp)	0.005266229
568974218	PREDICTED: Na(+)/H(+) exchange regulatory cofactor NHE-RF1	0.004976965
148688350	mCG128474	0.004976965
46592839	GRIP1 associated protein 1(Gripap1)	0.004976965
160707909	vasodilator-stimulated phosphoprotein(Vasp)	0.004976965
6755973	lin-7 homolog C (C. elegans)(Lin7c)	0.004976965
23503231	Ras association (RalGDS/AF-6) domain family (N-terminal) member 8(Rassf8)	0.004976965
31560316	G protein-coupled receptor 180(Gpr180)	0.004976965

148674121	syntrophin, acidic 1(Snta1)	0.003414382
115270960	BCL2-associated athanogene 3(Bag3)	0.003414382
13507620	retinoic acid induced 14(Rai14)	0.003414382
14198253	TNFAIP3 interacting protein 1(Tnip1)	0.003414382

Table S2 KEGG pathway enrichment analysis of YAP interactors

Term	Count	%	P-Value	Benjamini	Genes
Hippo signaling pathway	14	18.7	1.3E-13	6.5E-12	Mob1a, Patj, Tead3, Wwtr1, Wwc1, Amot, Mpp5, Ppp1ca, Trp53bp2, Ywhae, Ywhah, Ywhag, Ywhaz, Yap1
Tight junction	10	13.3	1.6E-08	4.0E-07	Patj, Amotl1, Cgn, Magi1, Magi3, Mpp5, Mpdz, Tjp1, Tjp2, Tjp3
Oocyte meiosis	5	6.7	2.0E-03	3.3E-02	Ppp1ca, Ywhae, Ywhah, Ywhag, Ywhaz
Epstein-Barr virus infection	6	8	3.8E-03	4.6E-02	Hspa1b, Nedd4, Ywhae, Ywhah, Ywhag, Ywhaz
mRNA surveillance pathway	4	5.3	1.1E-02	1.1E-01	Cpsf6, Cpsf7, Nudt21, Ppp1ca
Regulation of actin cytoskeleton	5	6.7	2.0E-02	1.6E-01	Arhgef7, Diaph1, Enah, Ppp1ca, Ppp1r12c
Cell cycle	4	5.3	2.3E-02	1.5E-01	Ywhae, Ywhah, Ywhag, Ywhaz
Endocytosis	5	6.7	4.7E-02	2.6E-01	Smurf2, Capza1, Hspa1b, Itch, Nedd4
Focal adhesion	4	5.3	8.1E-02	3.7E-01	Diaph1, Ppp1ca, Ppp1r12c, Vasp

Table S3 GoTerm_MF_Direct analysis of YAP interactors

Term	Count	%	P-Value	Benjamini	Genes
protein binding	39	52.0	2.1E-10	2.8E-8	Bag3, Patj, Arhgef7, Smurf2, Wbp2, Wwtr1, Amot, Amotl1, Amotl2, Capza1, Cgn, Diaph1, Dtnb, Enah, Hspa1b, Itch, Magi1, Magi3, Mpp5, Mpdz, Nedd4, Pkd2, Ppp1ca, Sept11, Stxbp4, Snta1, Sntb1, Sntb2, Tjp1, Tjp2, Tjp3, Ywhae, Ywhah, Ywhag, Ywhaz, Utrn, Uaca, Vasp, Yap1
actin binding	13	17.3	2.1E-9	1.4E-7	Fkbp15, Triobp, Capza1, Cgn, Diaph1, Enah, Snta1, Sntb1, Sntb2, Ywhah, Ywhag, Utrn, Vasp
cadherin binding involved in cell-cell adhesion	8	10.7	5.8E-5	2.6E-3	Bag3, Capza1, Cgn, Tjp1, Tjp2, Ywhae, Ywhaz, Vasp
protein domain specific binding	8	10.7	7.2E-5	2.4E-3	Lin7c, Mpp5, Tjp1, Tjp2, Ywhae, Ywhah, Ywhag, Ywhaz
profilin binding	3	4.0	6.7E-4	1.8E-2	Diaph1, Enah, Vasp
ion channel binding	5	6.7	8.7E-4	1.9E-2	Diaph1, Pkd2, Snta1, Ywhae, Ywhah

protein C-terminus binding	5	6.7	6.5E-3	1.2E-1	Mpdz, Nedd4, Tjp1, Tjp2, Yap1
protein complex scaffold	3	4.0	1.2E-2	1.8E-1	Wwc1, Magi1, Magi3
phosphoserine binding	2	2.7	1.4E-2	1.9E-1	Nedd4, Ywhae
calmodulin binding	4	5.3	2.7E-2	3.1E-1	Snta1, Sntb1, Sntb2, Tjp1
protein complex binding	5	6.7	3.8E-2	3.8E-1	Bag3, Carmil1, Ppp1ca, Ywhae, Ywhaz
poly(A) RNA binding	9	12.0	4.3E-2	3.9E-1	Cpsf6, Cpsf7, Diaph1, Nudt21, Sntb2, Ywhae, Ywhag, Ywhaz, Ubap2l
alpha-actinin binding	2	2.7	6.2E-2	4.9E-1	Magi1, Pkd2
proline-rich region binding	2	2.7	6.2E-2	4.9E-1	Nedd4, Yap1
PDZ domain binding	3	4.0	6.4E-2	4.7E-1	Lin7c, Snta1, Sntb1
SH3 domain binding	3	4.0	6.6E-2	4.6E-1	Enah, Trp53bp2, Vasp
protein kinase binding	5	6.7	6.8E-2	4.5E-1	Arhgef7, Kif13b, Ppp1r12c, Ywhaz, Utrn
identical protein binding	6	8.0	7.1E-2	4.4E-1	Smurf2, Amotl1, Amotl2, Pkd2, Trp53bp2, Ywhaz
actin filament binding	3	4.0	8.0E-2	4.7E-1	Triobp, Capza1, Utrn
mRNA binding	3	4.0	9.1E-2	4.9E-1	Cpsf6, C1qbp, Nudt21

Table S4 Parameter values for the biochemical model of YAP regulation

Symbol	Name	Value	Unit	Source
V_c	Volume of cytoplasm per cell	5450	μm^3	Measured on dataset reported previously (Morales-Navarrete *et al*, 2015)
V_n	Volume of all nuclei per cell	860	μm^3	Measured on dataset reported previously (Morales-Navarrete *et al*, 2015)
k_1	Max. flux of SENSOR activation	1	$1/\mu m^3$*d	free choice
K_{M1}	M-M const. of SENSOR activation	0.0008	$1/\mu m^3$	fit
k_2	Max. flux of SENSOR inactivation	2.02	$1/\mu m^3$*d	fit
K_{M2}	M-M const. of SENSOR inactivation	0.25	$1/\mu m^3$	fit
$k_{3,0}$	Factor of YAP synthesis rate	1.7	1/d	fit
k_4	YAP inactivation rate	0.19	1/d	fit
k_5	YAP activation rate	1	1/d	free choice
k_6	$_{inact}$YAP binding rate to SF	0.18	1/d	fit
k_7	$_{inact}$YAP unbin. rate from SF	1	1/d	free choice
k_8	YAP export rate from nucleus	1	1/d	free choice
k_9	YAP import rate into nucleus	0.17	1/d	fit
k_{10}	YAP degradation rate	1.8	1/d	fit
k_{11}	$_{inact}$YAP degradation rate	30	1/d	fit
K_{tot}	Conserved ([SENSOR] + [$_{inact}$SENSOR]) constrains the initial condition	5.5	$1/\mu m^3$	free choice

Supplementary Experimental Procedures

3D reconstruction and spatial analysis of BC network diameter

The BC network was reconstructed from 3D image stacks of CD13-stained tissue samples using the software MotionTracking as previously described (Morales-Navarrete *et al*, 2015; Meyer *et al*, 2017). To reconstruct 3D image stacks of CD13, DAPI and phalloidin-stained tissue samples, a tile of 2 x 1 image stacks was stitched to cover an entire CV-PV axis. Then, the CD13 and DAPI channels were aligned to the 2-photon (DAPI + phalloidin) channel and image intensities were normalized as previously described (Morales-Navarrete *et al*, 2015). CD13 images were segmented using a local thresholding algorithm (maximum entropy), segmented objects were corrected for artefacts using standard morphological operations (opening/closing) and the triangulation mesh of the segmented surfaces was generated by the cube marching algorithm. A representation of the skeletonized image was generated using a 3D graph describing the geometrical and topological features of the bile canaliculi network. To reconstruct the CV and PV, the intensity of the DAPI and phalloidin channels were added and the vessels were segmented from the inverse signal.

For spatial analysis of the BC diameter, the CV-PV axis was computationally divided into 20 equidistant zones and canaliculi were assigned to the individual zones according to their relative position χ to the CV and PV using the following equation:

$$\chi = \frac{\mathrm{d}_{CV}}{\mathrm{d}_{CV} + \mathrm{d}_{PV}} * 20$$

Where d_{CV} and d_{PV} are the distance of BC from CV and PV, respectively. The BC diameter was quantified in the xy-plane and determined as average per zone. The zones directly adjacent to the CV and PV (zone 1 and 20, ~ 1 cell layer) were excluded from the analysis.

Spatial analysis of nuclear YAP and PCNA and total YAP intensities within the CV-PV axis

Nuclear YAP and PCNA as well as total YAP intensities were quantified from 2 x 1 image tiles covering an entire CV-PV axis. For nuclear quantifications, nuclei were segmented by DAPI intensity thresholding using the Fiji software and non-parenchymal cells were excluded based on size and circularity. Using the MotionTracking software, the lobule axis was computationally divided into 10 isocentric zones and the spatial position of the nuclei was calculated as described above (3D reconstruction and spatial analysis of BC network diameter). For total YAP quantifications, the average image intensity per zone was determined. For all quantifications, the median of the mean nuclear YAP and PCNA or total YAP intensities was calculated per zone. For each timepoint of a time course, the mean nuclear intensity for each zone from 3-5 CV-PV axes was quantified.

Quantification of apical F-actin and pMLC density

IF images of CD13 and F-actin or pMLC, acquired within the PV area, were segmented based on the apical marker CD13 using the mean shift (Yizong Cheng, 1995) and the maximum entropy algorithms (Brink, 1996) implemented in the MotionTracking Software. To exclude BC lumens, intensity thresholding of the F-actin or pMLC intensity was additionally used. The mean F-actin or pMLC intensity was calculated per image and averaged for all images per time point. Within each condition (sham or PH), individual time courses were scaled to the mean value of all time courses by applying a scaling factor that was calculated as:

$$f_i = \frac{\sum_{j=1}^{N} y_{i,j} Y_j}{\sum_{j=1}^{N} Y_j^2}$$

Where $y_{i,j}$ is the intensity of the *i*-th curve in the *j*-th time point, Y_j is the mean intensity of all curves in the *j*-th time point and f_i is a scaling factor for the *i*-th curve, $Y_j = \frac{1}{K}\sum_{i=1}^{K} y_{i,j}$, N is the number of time

points, K is the number of the individual curves. The average intensity of all scaled time courses of one condition (sham or PH) was normalized to the untreated control (time point 0).

Segmentation of BC and quantification of BC membrane length and perimeter from EM images

To determine BC membrane length, BC membranes, including intra-luminal microvilli, were manually segmented from 10 x 10 EM image grids using the image analysis software Imod (http://bio3d.colorado.edu/imod). To estimate BC perimeter, the minimal enclosing ellipse of each BC segmentation was computationally determined as described previously (Todd & Yıldırım, 2007). On average, 55 BC were quantified per sample.

Quantification of the sub-apical and -basolateral gold particle density from immuno-EM images

The apical and basolateral plasma membranes were segmented manually from 6 x 6 EM image grids (~ 30 x 30 µm) using the Imod software. Gold particles within the area 200 nm below the plasma membrane were quantified (see Fig. S5B) and gold particle density (particles per area) was calculated. For each EM image grid, the average gold particle density of all basolateral and apical membranes was determined.

Mathematical model of YAP activation

Modeling strategy

We considered a linear array of hepatocytes along the central-portal axis of a liver lobule. The individual hepatocytes were assumed to respond independently from each other to the local strength of a mechanical stimulus by YAP activation, i.e. increased concentration of YAP in the nucleus as compared to the untreated control. The mechanical stimulus comprises stress and strain which are caused by increased intra-canalicular fluid pressure in response to fluid inflow driven by osmotic pressure that is proportional to the osmolyte concentration within the BC. We developed and coupled two sub-models

to describe the changes of osmotic pressure and the concomitant activation of YAP after PH. Sub-model 1 is a biophysics-based model to predict the local mechanical stress and apical membrane strain that result from the alteration of osmolyte (bile acid) load in the BC network after PH. It considers the spatial geometry of the BC within the CV-PV axis of the lobule. Sub-model 2 is a biochemistry-based model that predicts the cellular response of YAP to the local mechanical stress. Model parameters are set as reported in the literature (Morales-Navarrete *et al*, 2015) or fitted to data reported here.

Model definition

Sub-model 1 – Prediction of local mechanical stress at the apical domain of hepatocytes

Bile flow through BC is driven by both peristaltic contractions of bile canaliculi and osmotic pressure, resulting from actively pumped bile salts and other osmolytes (Meyer *et al*, 2017; Watanabe *et al*, 1991). Upon PH, with resection ratio r=62.8%±1.1% (mean and SEM) of liver mass without removal of the gall bladder, the total bile salt pool in the body is only marginally affected since intra-hepatic bile acids only account for 2-4% of the total bile acid pool (Setchell *et al*, 1997). The body also retains the capacity of intestinal bile acid reabsorption. However, it increases the bile acid load in the liver remnant, which needs to transport the full bile salt pool through a proportionally reduced BC network. This increases the bile salt secretion flux per canaliculus by analogy to experiments of intravenous bile acid injection (1). The concentration of osmolytes (c) within BC and consequently the osmotic pressure (p) have been shown to increase as the square root of the apical osmolyte secretion flux (Meyer *et al*, 2017; Ostrenko *et al*, 2019). Given that bile acids are themselves major osmolytes in bile (Anwer, 2004) and stimulate osmolyte secretion, we here approximate c as the bile acid concentration in BC. The pressure magnitude is considered to be linearly dependent on the osmolyte concentration (Mathias, 1985) while any spatial pressure dependencies are assumed as a common factor that cancels out in the ratio, see below. We

predict the fluid pressure *p(x)* within BC to increase relative to the pressure $p_0(x)$ of the control condition (sham operated mice) by the factor

$$\frac{p(x)}{p_0(x)} = \frac{c}{c_0} = \sqrt{\frac{100\%/(100\% - r)}{100\%/100\%}} = 1.64 \quad .$$

This increased intra-luminal pressure inflates the volume, and radius (a), of BC until it is counterbalanced by the Laplace pressure ($\tilde{p}$) exerted by the acto-myosin cortex. Hence, the apical hepatocyte membrane experiences mechanical strain γ_{strain} and the actin cortex mechanical stress γ_{stress}, together representing the mechanical stimulus γ.

This mechanical strain γ_{strain} of the apical membrane of each hepatocyte can be modeled as the ratio of half the BC circumferences upon PH and the control condition

$$\gamma_{strain}(x) = \frac{2\pi a(x)/2}{2\pi a_0(x)/2} = \frac{a(x)}{a_0(x)} \quad .$$

Towards the mechanical stress and assuming a cylindrical geometry of BC, the Laplace pressure is given by $\tilde{p} = \tilde{\gamma}\ \frac{1}{a}$ where $\tilde{\gamma}$ is the surface tension, or here cortical tension at the apical domain, that represents a mechanical stress (for brevity termed cortical tension in the following). Since Laplace and fluid pressure are equal at steady state, we obtained a prediction for the *relative* increase of cortical tension, here denoted $\gamma_{stress}(x)$, after PH as a function of position (*x*):

$$\gamma_{stress}(x) = \frac{\tilde{\gamma}(x)}{\tilde{\gamma}_0(x)} = \frac{p(x)}{p_0(x)} * \frac{a(x)}{a_0(x)} = \sqrt{\frac{100\%}{100\% - r}} * \frac{a(x)}{a_0(x)} \quad .$$

Note, the means of the measured values for *r*, *a*(*x*), a_0(*x*) possess small SEM (below 10%) and we therefore approximate the SEM of the calculated cortical tension $\gamma_{stress}(x)$ by propagating the SEM of the three measured quantities according to

$$SEM_\gamma(x) = \sqrt{\frac{100\%}{100\% - r} * \frac{a(x)}{a_0(x)}}$$

$$* \sqrt{\left(\frac{1}{2}\frac{100\%}{100\% - r}\right)^2 SEM_r{}^2(x) + \left(\frac{1}{a}\right)^2 SEM_a{}^2(x) + \left(\frac{1}{a_0}\right)^2 SEM_{a_0}{}^2(x)} \quad .$$

The predicted relative cortical tension levels of the bile canaliculi during regeneration are shown in Fig. S9A.

As a test of the predictive power of sub-model 1, we compared the predicted relative cortical tension to the measured relative pMLC levels (from Fig.2D) during regeneration. Since average pMLC levels were measured in the periportal 1/3 of the central-portal axis, they were compared to the averaged cortical tension values from the same spatial zones (Fig. S9B). Fig. S9B and a linear regression analysis reveal a high positive Pearson correlation (r=0.94) between the inferred cortical tension and pMLC levels, supporting sub-model 1. The diagonal line in Fig. S9B represents the linear curve fit $\frac{pMLC}{pMLC_0} = 1 + 0.62 * (\gamma_{stress} - 1)$.

The predicted cortical tension γ_{stress} and the measured membrane strain γ_{strain} both contribute to the total mechanical stimulus $\gamma = \alpha\,\gamma_{stress} + (1-\alpha)\,\gamma_{strain}$ which we use as input to sub-model 2 to predict the local response of YAP (Benham-Pyle *et al*, 2015; Fletcher *et al*, 2018). As both contributions to γ are proportional to $a(x)/a_0(x)$ and any pre-factors are constants, it suffices to study the dependency on one choice of the weighting parameter $0 \leq \alpha \leq 1$ and we have chosen α=1 here. Any choice α will yield identical results below, given the available data for the parameter estimation procedure (see below) and the freedom to rescale the parameter value k_1 in sub-model 2 which always occurs in a product with γ.

Sub-model 2 – Prediction of YAP activation by cortical tension

The model of YAP activation by mechanical stimulation γ is derived as follows and sketched in Fig. 7A. Solid (dashed) arrows in Fig. 7A denote reactions or transport steps (regulatory interactions), respectively. Depending on the position within the CV-PV axis, hepatocytes are exposed to different biliary pressures (Meyer *et al*, 2017) and thus experience spatially heterogeneous mechanical stimulation at the apical domain. We assumed that hepatocytes adapt to this spatially heterogeneous background mechanical stimulation under normal conditions. Further, we assumed that a relative increase of γ activates YAP through a combination of signaling cascade and cytoplasmic retention. The signaling cascade may depend on phosphorylation, as in the Hippo pathway, but alternative molecular states are possible, and can be accounted for, by an equivalent mathematical model (Grijalva *et al*, 2014; Loforese *et al*, 2017; Lu *et al*, 2018). YAP inactivation by the inactive form of a mechanosensor, here termed SENSOR, causes the cytoplasmic retention (by binding to cytoplasmic sequestration factors, here termed SF) or degradation of YAP, thus preventing its translocation into the nucleus. Based on this, the model considers five YAP regulatory mechanisms: YAP synthesis, degradation, (in)activation, cytoplasmic retention and nuclear-cytoplasmic shuttling. Further, we use $\gamma(x)$ as the mechanical stimulus that activates the mechanosensor.

Altogether, sub-model 2 comprises 2 sub-cellular compartments (cytoplasm and nucleus), 11 reactions as numbered in Fig. 7A and 6 variables for species concentrations that depend on the spatial position of the hepatocyte within the CV-PV axis and time after PH. Reactions 1 and 2 are modelled according to Michaelis-Menten rate laws, accounting for the limited amounts of upstream mechanosensors (parameters k_1, K_{M1} and k_2, K_{M2}, respectively). The mechanical stimulus $\gamma(x)$ is a factor of the maximum activation rate k_1. YAP synthesis is modeled as influx k_3. Within the lobule axis, hepatocytes are heterogenous with respect to their ploidy and nuclei number (Morales-Navarrete *et al*, 2015) as well as metabolic profile (Jungermann & Sasse, 1978). This may affect cellular YAP protein levels. To account for potential spatial differences in YAP protein synthesis, we quantified the total YAP intensity profile at

time point *t*=2d post PH as a proxy for relative (dimensionless) protein synthesis rate *s(x)* and inserted this experimentally measured spatial profile into the model equation for $k_3(x)=k_{3,0}*s(x)$.

All other reactions throughout the cytoplasm (numbers i=4-11) are modelled with simple mass action kinetics, each with a single rate constant k_i, see Table S4. The rate of the inactivation reaction 4 (Fig. 7A) was set proportional to the concentration of inactive mechanosensors $[_{inact}SENSOR]$. For transport between subcellular compartments of different volumes by reactions 8 and 9, scaling factors of volume ratios are derived from conservation of mass and introduced in the equations for cytoplasmic YAP ([YAP]) and nuclear YAP ([nYAP]).

Hence, the dynamics of the reaction network shown in Fig. 7A is modeled by 6 ordinary differential equations (ODE) for the 6 species' concentrations, as follows:

$$\frac{d[SENSOR]}{dt} = \frac{k_1 \cdot \gamma(x) \cdot [_{inact}SENSOR]}{K_{M1} + [_{inact}SENSOR]} - \frac{k_2 \cdot [SENSOR]}{K_{M2} + [SENSOR]}$$

$$\frac{d[_{inact}SENSOR]}{dt} = -\frac{k_1 \cdot \gamma(x) \cdot [_{inact}SENSOR]}{K_{M1} + [_{inact}SENSOR]} + \frac{k_2 \cdot [SENSOR]}{K_{M2} + [SENSOR]}$$

$$\frac{d[YAP]}{dt} = k_{3,0} \cdot s(x) - k_4 \cdot [_{inact}SENSOR] \cdot [YAP] + k_5[_{inact}YAP]$$

$$+ k_8 \cdot \frac{V_N}{V_C} \cdot [nYAP] - k_9 \cdot [YAP] - k_{10} \cdot [YAP]$$

$$\frac{d[_{inact}YAP]}{dt} = k_4 \cdot [_{inact}SENSOR] \cdot [YAP] - k_5[_{inact}YAP]$$

$$- k_6[_{inact}YAP] + k_7[_{inact}YAP_{SF}] - k_{11} \cdot [_{inact}YAP]$$

$$\frac{d[_{inact}YAP_{SF}]}{dt} = k_6 \cdot [_{inact}YAP] - k_7[_{inact}YAP_{SF}]$$

$$\frac{d[nYAP]}{dt} = k_9 \cdot \frac{V_C}{V_N} \cdot [YAP] - k_8[nYAP]$$

$$[YAP_{total}] = \frac{V_C}{V_C+V_N} \cdot \left([YAP] + [_{inact}YAP] + [_{inact}YAP_{SF}]\right) + \frac{V_N}{V_C+V_N}[nYAP]$$

The last algebraic equation provides the average total concentration of all YAP forms as an observable that can be compared to measured average YAP intensity data.

For model analysis, we considered two time scales, one fast (minutes to hours) of protein modifications and turnover, versus one slow (days) time scale of tissue growth. On the fast time scale, model simulations always converged to a unique stable steady state for a given mechanical stimulus. Assuming that the mechanical stimulus changes on the slow time scale, the state of the model adapted accordingly, rendering it a quasi steady state. To keep the model simple, we considered temporally constant and spatially uniform parameter values.

Parameter estimation

The model comprises 16 parameters. Of these, the compartment volumes of nuclei and cytoplasm, have previously been measured (Morales-Navarrete *et al*, 2015) and are set to the published values as referenced in Table S4. As revealed by the analytical model analysis (see below), five of these parameters affect the steady state solution only in parameter ratios of forward and backward rates of the four reversible reactions, hence individually these parameters are non-identifiable from given steady state data. We therefore set the value of five selected parameters to 1, see Table S4. The remaining 9 parameters were estimated by fitting the quasi steady state solution of the model to 120 independent data points from two observables on 6 experimental conditions (t=0d, t=0.8d, 1.5d, 2.0d, 3.0d, 5.0d) at 10 different spatial locations along the central-portal axis. We used a combination of Evolutionary Programming and Levenberg-Marquardt as global and local optimizers as implemented in the software Copasi (Hoops *et al*, 2006). Parameter optimization was performed in the software Copasi until convergence and results were confirmed by simulations in the software Morpheus (Starruß *et al*, 2014).

Model results

Analyzing the 6 ordinary differential equations at steady state, we predicted nuclear YAP levels as a function of mechanical stimulus $\gamma(x,t)$. We found a unique solution that represents a stable steady state:

$$[nYAP](\gamma) = \frac{V_C}{V_N} * \frac{k_9}{k_8} * \frac{k_3}{k_{10}} * \frac{1}{1 + \frac{2k_2 K_{M1} * {k_4}/{k_{10}}}{\left(1 + {k_5}/{k_{11}}\right) * f(\gamma)}}$$

$$[YAP_{total}](\gamma) = \frac{V_C}{V_C + V_N} * \frac{k_3}{k_{11}} * \left(1 + \frac{k_6}{k_7}\right) + \frac{V_C}{V_C + V_N} * \frac{k_3}{k_{10}} * \frac{1 + \frac{k_9}{k_8} - \frac{k_{10}}{k_{11}} * \left(1 + \frac{k_6}{k_7}\right)}{1 + \frac{2k_2 K_{M1} * {k_4}/{k_{10}}}{\left(1 + {k_5}/{k_{11}}\right) * f(\gamma)}}$$

$$f(\gamma) = \gamma k_1 \left(1 + \frac{K_{M2}}{K_{tot}}\right) + k_2 \left(\frac{K_{M1}}{K_{tot}} - 1\right) + \sqrt{\left(\gamma k_1 \left(1 + \frac{K_{M2}}{K_{tot}}\right) + k_2 \left(\frac{K_{M1}}{K_{tot}} - 1\right)\right)^2 - 4(\gamma k_1 - k_2) k_2 \frac{K_{M1}}{K_{tot}}}$$

This closed analytical form of the steady state solution allowed us to understand the role of individual parameters in the response of hepatocytes and guided the parameter estimation strategy (see above). Specifically, given steady state data for nuclear and total YAP levels, only a subset of the parameters is individually identifiable while others only affect the solution through ratios of parameters and were chosen for computational convenience (see Table S4).

Employing this spatially and temporally constant parameter set (Table S4) for all 120 available data points, the model predicted nuclear (see Fig. S9C) and total (see Fig. S9D) YAP profiles from the position- and time-dependent mechanical stimulus $\gamma(x,t)$ and the position-dependent but temporally constant proxy for protein synthesis (see Fig. S9D, red curve). Overall, we observe a very good match between the confidence intervals of data and model prediction, both indicated by SEM intervals in Figs. S9C and S9D, where the calculated SEM intervals of $\gamma(x,t)$ (see Fig. S9A) have been propagated to SEM intervals for YAP. Spatial fluctuations in the model prediction are ultimately a response to spatial fluctuations in the

experimentally measured radius profiles that are used to predict $\gamma(x,t)$. Just for the last time point, t=5d, the model prediction overestimates for both nuclear and total YAP levels. The model source code is provided as supplementary file.

Having established the mechanistic model of YAP turnover, activation and subcellular translocation, we next asked how sensitive the YAP response is with respect to the mechanical stimulus. To study this question, we inserted a spatially averaged protein synthesis rate (s=<*s(x)*>=1.5) as a constant and considered γ as a control parameter. The resulting stimulus-response curve (see Fig. 7B, black solid line) reveals a plateau around the normal unstressed condition $\gamma < 1.8$ followed by a sigmoidal part with a slope of approximately 500% and a plateau at high stimulus levels. Note, the scatter around the theoretical dose-response curve is attributable to the spatial variation in protein synthesis rate that was not considered in the spatially averaged model prediction for constant s=<*s(x)*>.

Such a sigmoidal stimulus-response curve of nuclear YAP levels ensures that normal (e.g. diet-triggered) fluctuations in biliary fluid pressure and hence cortical tension are tolerated and do not mount a regenerative response while a stronger pressure increase robustly activates YAP in a switch-like manner.

Statistics

For quantification of BC diameter (Fig. 1B, 6B), BC perimeter and membrane length (Fig. 1D, 1E), apical F-actin and pMLC density (Fig. 2B, 2D) as well as nuclear YAP and PCNA intensity (Fig. 3C, 3D, 6D), *n* represents the number of mice per condition or timepoint. For quantification of sub-apical and -basolateral YAP density from EM images (Fig. S5C), *n* represents the number of EM image grids analyzed. For quantification of nuclear and cytoplasmic intensity of YAP from IF images of hepatocyte cultures (Fig. 5B, 5E, S7A), pMLC levels from Western blot (Fig. S7B, C) and gene expression levels (Fig. S7d), *n* represents the number of experiments. Measurements are given as mean +/- standard error of the mean (s.e.m.; Fig. 1B, 2B, 2D, 3C, 3D, 5B, 5E, 6B, 6D, S7, S9) or box-whisker plot with median, 25-75

quartiles and minimum/maximum error bars (Fig. 1D, 1E, S5C). The significance of the difference of two conditions was estimated by Student's t-test. To calculate the significance of the difference of two spatial or temporal profiles a and b (Fig. 1B, 2B, 2D, 3C, 3D, 6B, 6D), the normalized differences Δ_i of two values a_i and b_i was calculated using

$$\Delta_i = \frac{(a_i - b_i)}{\sqrt{{\sigma_{a_i}}^2 + {\sigma_{b_i}}^2}}$$

where *i* is the zone index within the CV-PV axis or time point of a time course. The Student's t-test was applied assuming as null hypothesis that the mean difference is 0. P-values in Figures are represented by asterisks, *, p ≤ 0.05; **, p ≤ 0.01; ***, p ≤ 0.001; ****, p ≤ 0.0001; ns., p > 0.05.

Software

The MotionTracking software (Morales-Navarrete *et al*, 2015) (http://motiontracking.mpi-cbg.de) was used for analysis and 3D reconstructions of IF images as well as statistical analysis. Imod (http://bio3d.colorado.edu/imod) was used for manual segmentation of EM images. GraphPad (GraphPad Software, Inc.) was used for graphical representations. Fiji (Schindelin *et al*, 2012) was used for background subtraction of IF images, image visualization and Western blot quantifications. Copasi (Hoops *et al*, 2006) was used for parameter estimation in the mathematical sub-model 2. Morpheus (Starruß *et al*, 2014) was used to simulate and analyze the coupled mathematical models. MASCOT software (Matrix Science, London, UK) was used to identify proteins from peptide sequences. Scaffold software (Proteome Software Inc., Portland, US) was used to validate MS/MS-based protein identifications. MaxQuant software was used to quantify relative abundance of proteins.

Supplemental References

Anwer MS (2004) Cellular regulation of hepatic bile acid transport in health and cholestasis. *Hepatology* **39:** 581–590

Benham-Pyle BW, Pruitt BL & Nelson WJ (2015) Mechanical strain induces E-cadherin-dependent Yap1 and -catenin activation to drive cell cycle entry. *Science* **348:** 1024–1027

Brink AD (1996) Using spatial information as an aid to maximum entropy image threshold selection. *Pattern Recognit. Lett.* **17:** 29–36

Cheng Y (1995) Mean Shift, Mode Seeking, and Clustering. *IEEE Trans. Pattern Anal. Mach. Intell.* **17:** 790–799

Fletcher GC, Diaz-de-la-Loza M-C, Borreguero-Muñoz N, Holder M, Aguilar-Aragon M & Thompson BJ (2018) Mechanical strain regulates the Hippo pathway in *Drosophila*. *Development* **145:** dev159467

Grijalva JL, Huizenga M, Mueller K, Rodriguez S, Brazzo J, Camargo F, Sadri-Vakili G & Vakili K (2014) Dynamic alterations in Hippo signaling pathway and YAP activation during liver regeneration. *Am. J. Physiol. - Gastrointest. Liver Physiol.* **307:** G196–G204

Hoops S, Sahle S, Gauges R, Lee C, Pahle J, Simus N, Singhal M, Xu L, Mendes P & Kummer U (2006) COPASI--a COmplex PAthway SImulator. *Bioinformatics* **22:** 3067–3074

Jungermann K & Sasse D (1978) Heterogeneity of liver parenchymal cells. *Trends in Biochemical Sciences* **3:** 198–202

Loforese G, Malinka T, Keogh A, Baier F, Simillion C, Montani M, Halazonetis TD, Candinas D & Stroka D (2016) Impaired liver regeneration in aged mice can be rescued by silencing Hippo core kinases MST1 and MST2. *EMBO Mol. Med.* **9:** 1–15

Lu L, Finegold MJ & Johnson RL (2018) Hippo pathway coactivators Yap and Taz are required to coordinate mammalian liver regeneration. *Exp. Mol. Med.* **50:** e423

Mathias RT (1985) Epithelial water transport in a balanced gradient system. *Biophys. J.* **47:** 823–836

Meyer K, Ostrenko O, Bourantas G, Morales-Navarrete H, Porat-Shliom N, Segovia-Miranda F, Nonaka H, Ghaemi A, Verbavatz JM, Brusch L, Sbalzarini I, Kalaidzidis Y, Weigert R & Zerial M (2017) A Predictive 3D Multi-Scale Model of Biliary Fluid Dynamics in the Liver Lobule. *Cell Syst.* **4:** 277–290.e9

Morales-Navarrete H, Segovia-Miranda F, Klukowski P, Meyer K, Nonaka H, Marsico G, Chernykh M, Kalaidzidis A, Zerial M & Kalaidzidis Y (2015) A versatile pipeline for the multi-scale digital reconstruction and quantitative analysis of 3D tissue architecture. *eLife* **4:** 1–29

Ostrenko O, Hampe J & Brusch L (2019) Wet-tip versus dry-tip regimes of osmotically driven fluid flow. *Sci. Rep.* **9:** 4528

Schindelin J, Arganda-Carreras I, Frise E, Kaynig V, Longair M, Pietzsch T, Preibisch S, Rueden C, Saalfeld S, Schmid B, Tinevez J-Y, White DJ, Hartenstein V, Eliceiri K, Tomancak P & Cardona A (2012) Fiji: an open-source platform for biological-image analysis. *Nat. Methods* **9:** 676–682

Setchell KDR, Rodrigues CMP, Clerici C, Solinas A, Morelli A, Gartung C & Boyer J (1997) Bile Acid Concentrations in Human and Rat Liver Tissue and in Hepatocyte Nuclei. *Gastroenterology* **112:** 226–235

Starruß J, de Back W, Brusch L & Deutsch A (2014) Morpheus: a user-friendly modeling environment for multiscale and multicellular systems biology. *Bioinformatics* **30:** 1331–1332

Todd MJ & Yildirim EA (2007) On Khachiyan's algorithm for the computation of minimum-volume enclosing ellipsoids. *Discrete Appl. Math.* **155:** 1731–1744

Watanabe N, Tsukada N, Smith CR & Phillips JM (1991) Motility of bile canaliculi in the living animal: Implications for bile flow. *J. Cell Biol.* **113:** 1069–1080
